# Relationship Between Rectal Temperature and Infrared Temperatures of a Goat’s External Body Surfaces

**DOI:** 10.3390/ani16091416

**Published:** 2026-05-06

**Authors:** Md. Niamot Ali, Abdulla Al Mamun Bhuyan, Mst. Ishrat Zerin Moni, Mst. Farzana Aktar, Md. Arafat Hossain, Dipa Ray, Mehedy Hasan Pranto, Md. Mahabubur Rashid, Jashim Uddin

**Affiliations:** Department of Veterinary and Animal Sciences, University of Rajshahi, Rajshahi 6205, Bangladesh

**Keywords:** rectal thermometry, infrared thermography, health assessment, goats, temperature measurement, eye IRT

## Abstract

Measuring rectal temperature is a common method for assessing the body temperature in animals in response to changes in physiology, health, and emotions. However, non-invasive infrared thermography is a more advanced technique that reduces the stress associated with restraining animals and minimises risks related to cross-contamination and disease transmission. The present study investigated the association between rectal temperature (RT) and infrared temperature (IRT) of the goat’s external body surfaces. A total of one hundred thirteen (113) goats were considered for this study, and those that showed signs of health deviations were excluded from the evaluation. Thus eighty-four (84) goats were used for the study. We recorded the RT of each goat, as well as the maximum (M) and average (A) IRT of the forehead, right and left ears, right and left eyes, right and left coronary bands of the forelimbs, right and left shoulders, and the mucous membrane of the mouth, simultaneously. The descriptive statistics revealed that the maximum IRT values were higher than RT, except for the right coronary band, left coronary band, right shoulder, and left shoulder. Pearson correlation showed RT has a positive association with all IRT measures, including maximum and average values. The highest association was observed between RT and right eye maximum IRT (REM) (r = 0.63). In addition, the regression analysis showed RT and REM have a moderate correlation (S = 0.36 and R^2^ = 39.6%). In conclusion, the maximum IRT of the right eye could be used as a non-invasive measure of body temperature in goats. More research is needed to investigate the potential of eye IRT towards automated recording of body temperature in goats.

## Simple Summary

Rectal temperature is commonly used to assess animal physiology, health, and emotional states. However, measuring rectal temperature is associated with restraining/handling stress, increases the chances of risks related to cross-contamination of infectious disease, and overall poor welfare. Recently, infrared thermography has been gaining popularity to estimate body temperature in animals, but it is still limited in goats. Therefore, the present study investigated the association between rectal temperature and infrared temperature of the external body surfaces of goats. In the present study, if any of the goats showed signs of health deviations, they were excluded from the evaluation. The correlation between rectal temperature and infrared temperature of the goat’s external body surfaces showed a positive association, particularly the association between right eye maximum infrared temperature and rectal temperature, which was also supported by the relevant association in the regression analysis. Therefore, the maximum infrared temperature of the right eye could be used as a non-invasive measure of body temperature in goats.

## 1. Introduction

Body temperature is a key physiological parameter to assess animal health. For example, any deviation in core body temperature indicates the early clinical signs of stress or disease in animals [1]. Conventionally, rectal thermometry has been widely practised for measuring body temperature in animals to assess their health status [1,2]. However, measuring the rectal temperature requires physical restraint and close contact of animals, which can cause stress, discomfort, and potential injury to both animals and handlers [3,4]. It also increases risks related to cross-contamination and disease transmission [5]. Overall, rectal thermometry poses poor welfare, and it may even alter physiological responses in animals [6].

Therefore, researchers have been investigating approaches to measure the body temperature of animals using diverse methods such as placing data loggers in different anatomical positions of animals [7,8]. The application of thermal data loggers could measure and record animal body temperature over time without handling the animal manually but sometimes may require insertion of the logger into the animal’s body by surgical means or a secure external attachment [7]. In addition, the main challenges are keeping the thermal data loggers in place on the animal body and retrieving them, which often requires another invasive procedure [7,8]. Thus, this method is also not appropriate for commercial use.

Recently, the application of infrared thermal sensors has been widely applied to assess the changes in temperature of the animal body and link it with fear, stress, anxiety, inflammation, and diseases [9]. These temperature differences are displayed as images known as thermograms, which makes the technique non-invasive and suitable for automated use. It can detect emitted thermal radiation as a temperature from the external body surfaces in animals, including eyes, limbs, foreheads, shoulders, and mucous membranes, and this emitted thermal radiation reflects changes in body temperature [10]. Infrared thermography is a more advanced technique that reduces the discomfort and stress associated with handling and restraining animals [4]. Additionally, infrared thermography is a fast and non-contact technique that can detect changes in the body temperature of animals due to diseases, changes in the environment, and/or stress in real time by monitoring changes in subsurface blood flow based on the detection of emitted infrared radiation from the body surface [11,12]. However, accurate measurement of external body surface temperature through infrared thermography requires minimising intrinsic factors such as skin and hair colour, emissivity, movement, health, and feeding time, as well as extrinsic factors including environmental temperature, humidity, radiation, wind speed, camera distance, and angle [13,14]. Establishing a validated relationship between rectal temperature and IRT of different body surfaces measured by an infrared thermal camera is essential for assessing its practical usefulness to evaluate animal welfare and health. There are many methods for developing a rectal temperature assessment model based on the IRT of different body surfaces in animals [1]. In the previous studies, simple linear regression analysis [15,16], multiple linear regression analysis [17], and stepwise linear regression analysis [1,18] were employed to develop a rectal temperature assessment model from IRT. Previous studies in equine [2], cattle [19,20,21], buffalo [22], and sheep [23] have reported varying degrees of correlation between rectal temperature and infrared temperature of different body surfaces, but the available data for goats are still limited [1,24]. Therefore, the current study aimed to investigate the association between rectal and infrared temperature of the goat’s external body surfaces to overcome the confounding factors associated with other measures of body temperature, such as rectal temperature.

## 2. Materials and Methods

The animal study protocol was approved by the Institutional Animal, Medical Ethics, Biosafety, and Biosecurity Committee (IAMEBBC of the Institute of Biological Sciences, University of Rajshahi, Bangladesh (Protocol code: 35/320(47)/IAMEBBC/IBSc), and the date of approval was 30 January 2025).

### 2.1. Study Population

The experiment was carried out on goats reared in three different communities, namely Talaimari, Meherchondi, and Panchabati in Rajshahi district, Bangladesh, in August of 2025. The goats included in the study were Black Bengal goats: black, grey, or black-and-white coat colour, with most of them in black. A total of one hundred thirteen goats were initially included for this study, regardless of their breed, age, sex, or body weight. Goats that showed signs of health deviations—fever, nasal discharge, cessation of rumination, dehydration, salivation, injury, facial grimaces, and/or soft or watery faeces—were excluded from the evaluation; thus, eighty-four goats, which were in good health, were used for the study. The included goats were rearing under a semi-intensive farming system: grazing freely for 5–6 h daily. These goats were commonly provided with green grass, jackfruit leaves, steamed rice or steamed broken rice, wheat, wheat bran, rice polish, maize bran, pulse husk, and drinking water. No medication, including antibiotics or internal or external antiparasitic agents, was administered during the study period. However, information on medication use prior to the study was not recorded, and therefore, prior administration cannot be evaluated or considered. Water was provided ad libitum, and the animals had free access to a consistent water source, either supplied tap water or tubewell water, stagnant water, or river water as well.

### 2.2. Infrared Thermography

**Figure 1:**
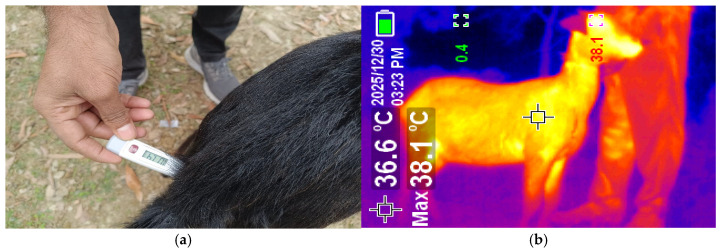
Illustrates the procedure of taking rectal temperature and infrared thermal temperature in a goat. (**a**) Taking rectal temperature using a digital thermometer. (**b**) Infrared thermogram of the right shoulder of a goat. The maximum and average IRT were extracted from the thermal images and captured the targeted body parts individually from a distance of 0.5 m in a straightforward manner. Focused body parts are shown in the picture with average (black-square-targeted position indicator), and maximum IRT (Max) values as well.

The evaluation procedure was performed in the evening, from around 5.00 to 6.30 pm. Whenever direct sun exposure to the goats during the study was avoided to prevent any potential misinterpretation of the results. At first, each goat was handled gently by one person, and thermograms were captured by another person from a 0.5 m distance (lens to goat’s forehead, ears, eyes, coronary band of forelimbs, shoulders, and mucous membrane of the mouth) (Figure 1b).

**Figure 2:**
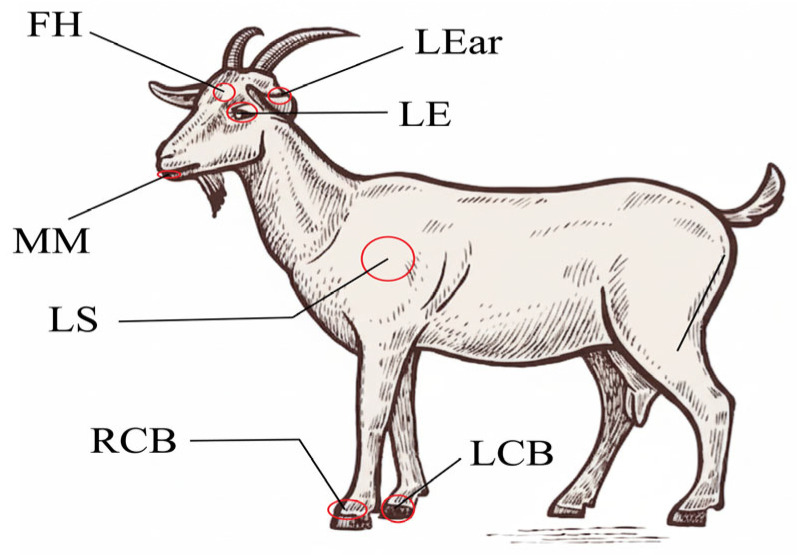
Experimental configuration showing the infrared temperature (IRT) measurement points on the external body surfaces of the goats. Here, FH = forehead, LEar = left ear, LE = left eye, MM = mucous membrane of the mouth, LS = left shoulder, RCB = right coronary band, and LCB = left coronary band.

IRT of external body surfaces (forehead, ears, eyes, coronary band of forelimbs, shoulders, and mucous membrane of mouth) was taken by using an infrared thermal camera (Uni-T Uti165H Professional Thermal Imager, Uni-Trend Technology Co., Ltd., Dongguan City, Guangdong Province, China) [Figure 2]. The camera could measure a temperature range from 30 to 45 °C with a resolution of 320 × 240 pixels and accuracy: ±0.5 °C. The infrared thermal camera was calibrated using the manufacturer’s factory calibration settings prior to data collection and calibrated at 25 °C ambient temperature, and the emissivity value was adjusted to 0.98. However, the camera was not specifically calibrated for relative humidity, as measurements were obtained at a short distance under relatively stable environmental conditions. The thermograms were obtained in a straightforward manner from all selected body surfaces individually, as homogeneously as possible. Surface temperatures were directly read from the thermal images displayed and captured by the camera, and no additional software was used for temperature extraction.

### 2.3. Rectal Temperature Recording

Rectal temperature of the goat was recorded by using a digital thermometer (Rossmax TG380 Flexible Thermometer, Rossmax, Taipei, Taiwan) just after capturing infrared thermal images of each goat [Figure 1a] by the same person who captured the thermal images of the same goat. The thermometer has a measurement range from 32.0 °C to 42.9 °C, with an accuracy of ±0.1 °C. The thermometer was inserted into the rectum for a length of about 2 cm at a 45° angle to ensure contact with the lateral rectal mucosa. To prevent cross-contamination, the rectal thermometer was disinfected after each animal’s measurement using a mixture of ethanol 80%, glycerol, hydrogen peroxide, and purified water (Germnil Hand Sanitizer, BMTF Limited, Gazipur, Bangladesh). This rectal thermometer provides results within 10 s and sounds an audible alarm when the maximum temperature is reached.

### 2.4. Recording Environmental Temperature and Relative Humidity

Environmental factors such as environmental temperature and wind speed were recorded onsite for the experimental period using a UNI-T Wind Speed Meter UT363BT (Uni-Trend Technology Co., Ltd., Dongguan City, Guangdong Province, China), and the humidity of the experimental area was recorded from the internet (https://www.accuweather.com/) to avoid error in the interpretation of thermal images. The Temperature–Humidity Index (THI) was calculated by using the formula: THI = (1.8E^T^ °C + 32) − [(0.55 − 0.0055RH) (1.8E^T^ °C − 26)], where E^T^ °C represents environmental temperature (°C) and RH represents the relative humidity (%) [25].

### 2.5. Statistical Analysis

All data were taken in the data collection sheet individually and input into Minitab (v2022) (Minitab, LLC, State College, PA, USA). Descriptive statistics of body temperatures were presented as mean, standard deviation, and range. The normality test for IRT of different body surfaces of goats was checked, and outliers were removed to obtain normally distributed data according to the Grubb’s Test (*p* > 0.05).

Data were used to evaluate the relationship between the RT and IRT of different body surfaces of the goats. Analytically, a paired *t*-test was used to assess differences between the maximum and average IRT of paired body surfaces individually. Finally, the correlations between RT and IRT were determined using the Pearson correlation test and regression analysis. Correlations with coefficients > 0.75 were classified as high, those between 0.36 and 0.70 as moderate, and those <0.35 as low [26]. The variations in different parameters were considered significant when the *p*-value was <0.05.

## 3. Results

As shown in Table 1, the descriptive statistics revealed that the maximum infrared temperature (IRT) of the body surfaces was higher than the rectal temperature (RT) with the exception of the IRT of the right coronary band, left coronary band, right shoulder, and left shoulder. In addition, the average difference in the maximum IRT of RE, LE, FH, RCB, LCB, REar, LEar, RS, LS, and MM compared to the RT was 1.6 °C, 1.7 °C, 0.5 °C, −0.8 °C, −0.8 °C, 0.5 °C, 0.7 °C, −0.2 °C, −0.1 °C, and 2.1 °C, respectively.

The descriptive statistics indicate that the IRT of the external body surfaces of goats had a higher maximum IRT in their mucous membrane of the mouth (41.1 ± 0.89 °C) and a lower maximum IRT in their right (38.2 ± 1.07 °C) and left (38.2 ± 1.05 °C) coronary band of the forelimb [Table 1]. Similarly, the IRT of the external body surfaces of goats has a higher mean average IRT in their mucous membrane of the mouth (39.8 ± 1.23 °C), and a lower mean average IRT in their left coronary band of the forelimb (37.0 ± 0.48 °C) [Table 1].

As shown in Table 2, a paired *t*-test comparing the IRT of different pairs of body surfaces in goats revealed that the maximum and average IRT of the eye coronary band of forelimbs did not differ due to their left or right body position of the same body surfaces. Similarly, the average IRT of ears and the maximum IRT of shoulders did not differ [Table 2]. However, the maximum IRT of the right and left ear and the average IRT of the right and left shoulder differed due to the left or right body position of the same body surfaces.

The correlations between the physiological response RT and the IRT of REM, LEM, FHM, RCBM, LCBM, REarM, LEarM, RSM, LSM, and MMM with a confidence interval of 95% had a positive correlation (*p* = 0.000) with the RT, with a Pearson correlation coefficient of 0.63, 0.55, 0.59, 0.43, 0.56, 0.57, 0.45, 0.43, 0.51, and 0.54, respectively (Table 3). The highest association was observed between REM and RT (r = 0.63). In addition, the association between FHM and RT (r = 0.59) and REarM and RT (r = 0.57), respectively, showed potential.

The correlations between the physiological response RT and the IRT of REA, LEA, FHA, RCBA, LCBA, REarA, LEarA, RSA, LSA, and MMA with a confidence interval of 95% had a significant positive correlation (*p* < 0.005) with the RT, with a Pearson correlation coefficient of 0.59, 0.55, 0.47, 0.46, 0.45, 0.48, 0.54, 0.38, 0.38, and 0.48, respectively (Table 4). The highest association was observed between REA and RT (r = 0.59). In addition, the association between LEA and RT (r = 0.55) and LEarA and RT (r = 0.54), respectively, showed potential.

**Figure 3:**
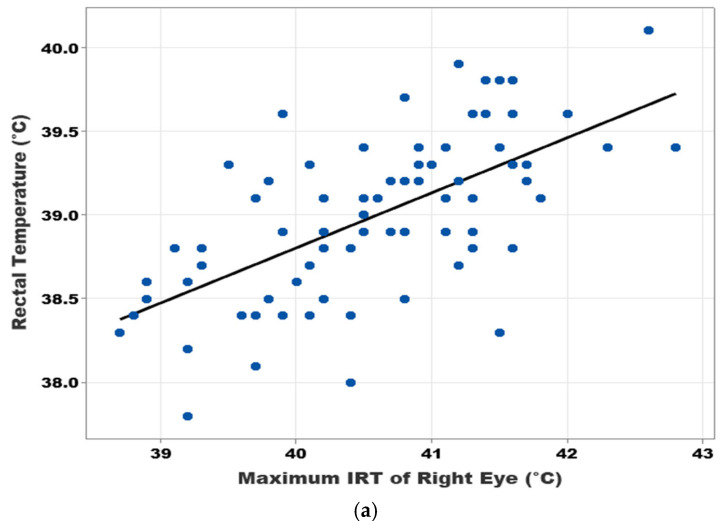

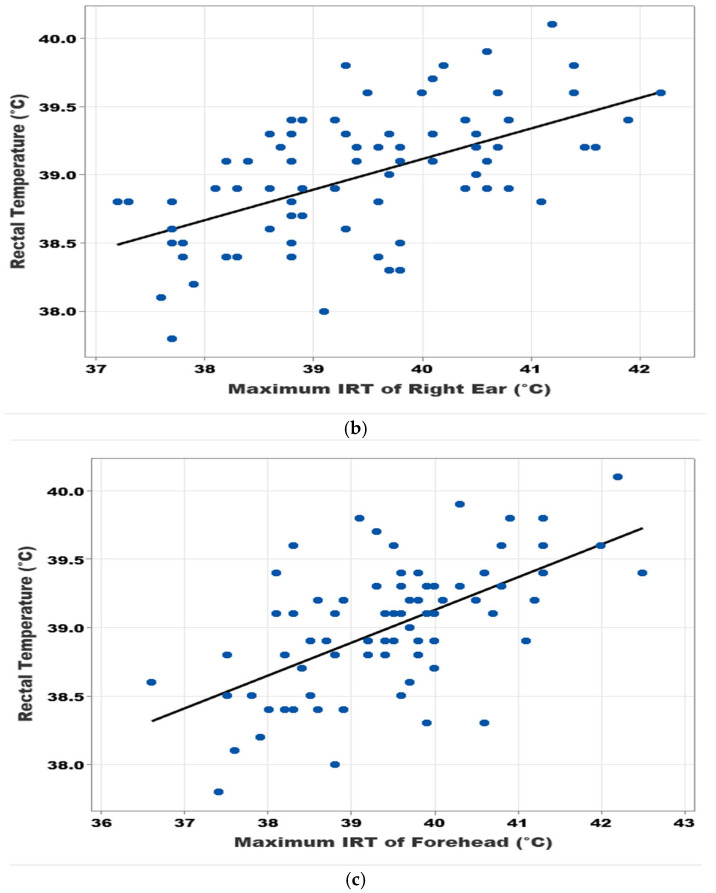
Illustrates the relationship between the rectal temperature (RT) and the maximum infrared temperature (IRT) of the right eye: RE (**a**), right ear: REar (**b**), and forehead: FH (**c**).

Here, S and R.Sq (R^2^) indicate the standard error of the regression and the coefficient of determination, respectively. In Figure 3a–c, the X-axis represents the rectal temperature of goats, and the Y-axis represents the REM, REarM, and FHM of goats, respectively. The relationship between the rectal temperature (RT) and the maximum IRT of the right eye (REM), right ear (REarM), and forehead (FHM): the standard error of the regression and coefficient of determination for these models are (S = 0.36, and R^2^ = 39.6%), (S = 0.39, and R^2^ = 31.9%), (S = 0.38, and R^2^ = 35.1%), respectively. In addition, the RT can be represented by the following mathematical expression: RT  ≅ 25.67 + 0.3283 REM, RT  ≅ 30.16 + 0.2239 REarM, and RT  ≅ 29.56 + 0.2392 FHM with insignificant mean errors.

## 4. Discussion

This study aimed to find out the association between rectal temperature (RT) and the infrared temperature (IRT) of different body surfaces of goats. We found the association between rectal temperature and IRT of the goat’s external body surface is positive. The highest association was between the maximum IRT of the right eye and rectal temperature, though there was no statistical difference between the IRT of right and left eye values.

Descriptive statistics revealed that the mean value of maximum IRT values of the right eye, left eye, forehead, right ear, left ear, and mucous membrane of the mouth was higher than the corresponding rectal temperature (RT) of the goats. In addition, the mean value of average IRT values of the right eye, left eye, and mucous membrane of the mouth was also higher than the mean RT of the goats. A previous study also reported similar findings that the maximum IRT is relatively higher than RT in goats [24]. Particularly, the maximum IRT is based on a single pixel and records the highest temperature reading in the targeted animal body surfaces, whereas the average IRT shows the value of all pixels averaged [27]. In fact, the distance between the thermal camera and animal body surfaces can also affect the reading, and a shorter distance causes less scatter of emitted infrared rays from the animal’s body surfaces and increases the value of the measured reading [28]. Previous studies reported that the mean IRT [(maximum IRT + average IRT)/2] of different body surfaces is lower than the RT in sheep [23], goats [1], buffaloes [22], fattening rabbits [29], and anaesthetized dogs [30]. Our study reported similar findings that the mean IRT of different body surfaces of goats was lower than the RT, except for the right eye, left eye, and mucous membrane of the mouth. Especially, several studies have reported that the maximum IRT of external body surfaces more effectively detects diseases and emotional states of an animal, such as fear, pain, and stress, than the value of the average or minimum IRT, and it is less affected by ambient temperature, resulting in lower variability [31,32,33].

The relatively higher IRT values observed in this study might be attributed to the shorter distance between the infrared camera and the goats’ body surface (0.5 m) as well as the coat colour of the goats. In the present study, the majority of the goats had black coats, with some exhibiting grey or black-and-white coloration. Previous studies recorded IRT measurements from a distance of 1 m [1,22,29] or 0.5 m [30] but did not report the coat colour of the animals. Coat colour is known to influence infrared temperature, as black hair has higher emissivity in the visible wavelength and absorbs and emits more solar energy than lighter hair, resulting in higher infrared temperatures [34]. Therefore, the predominance of black-coated Black Bengal goats in this study might have contributed to the higher IRT values observed. While previous research did not mention the coat colour of the studied animals.

In fact, body temperature is mostly influenced by environmental changes, and the temperature of external body surfaces of animals is an important index to evaluate heat stress [35]. Even the IRT of different body surfaces of an animal is very sensitive to environmental temperature, and increasing the environmental temperature influences the changes in IRT of animal body surfaces [24]. Especially, the mucous membrane and eyes are highly sensitive organs because environmental changes can trigger thermal responses in these organs. During heat stress, an increase in core body temperature triggers greater peripheral blood flow to release excess heat [36], resulting in higher heat loss from body surfaces such as the eyes and mucous membranes of the mouth. Thus, higher environmental temperature increases the body’s external and internal temperature in animals [37,38]. For example, in colder environments, blood flow is directed to the internal organs, resulting in a decrease in eye temperature [39]. Finally, the higher environmental temperature and higher relative humidity of our study area might have also influenced the fact that our IRT of different external body surfaces of goats was relatively higher.

Due to the environmental temperature, relative humidity, and temperature-humidity index being relatively higher in the present study, the measured values were 32.7 ± 1.64 °C, 82.2 ± 3.55%, and 87.5 ± 2.52, respectively. While the environmental temperature and relative humidity were around 20–29 °C, and 55–65% in the previous studies, respectively [1,21,28]. In addition, THI values ≤ 72, =73–78, and ≥80 indicate that comfortable, mild heat stress, and severe heat stress in goats, respectively [40]. Therefore, it is demonstrated that the environmental parameters and IRT of different external body surfaces have a positive correlation in the present study. As the environmental parameters were higher in this study, which could influence the maximum IRT of the external body surfaces of goats, it was higher than their RT.

A previous study included sheep, where they were reared in intensive farming without access to pasture land or free grazing, and the sheep were provided with cereal straw, maize cane silage, artichoke by-product, and commercial feed supplements [23]. In contrast, the goats included in this study were reared under a semi-intensive system without the use of commercial feed supplements. They were provided with conventional concentrate feeds and allowed free scavenging for approximately 5–6 h per day. However, the quantity of feed supplied was not standardised, and management practices might be varied among farmers. Consequently, both the type and amount of feed provided might have differed across farms. In addition, animal sex may have influenced physiological responses [41]; however, this study did not evaluate the sex and age-specific relationship between IRT and RT.

The present study reveals that the IRT of different body surfaces showed a positive association with RT in goats. A previous study reported similar findings, but the IRT of different external body surfaces in sheep shows moderate or low correlations with rectal temperature [23]. This study also investigated the correlation of the maximum and average IRT of paired body surfaces of goats. The findings indicated that there was no difference between the IRT of the right and left eyes in goats. The present study also demonstrated that the maximum and average IRT of all included pair body surfaces did not show any statistical difference, except for the maximum IRT of REar and LEar and the average IRT of RS and LS of goats. A previous study in buffaloes showed that the IRT of the left and right eyes does not have a significant difference between them, but the right eye temperature is relatively higher than the left eye [22]. In addition, another study revealed that left eye temperature is lower than the right eye during stress conditions, and the right eye shows higher sensitivity [13,32,42]. However, other studies in cats showed that there is no statistical difference between the IRT of the right and left eyes [15]. Similarly, Uddin et al. (2019) and Idris et al. (2024) demonstrate that there is no difference in the left and right eye IRT of cows under exposure to the environmental stressors [12,43]. Goats are well-known small ruminants, and they can tolerate high environmental temperatures due to their ability to reduce metabolic activities, preventing body temperature from increasing during heat stress, reducing the risk of death for the animals, but causing a fall in productivity [24]. In addition, the highest association in their physiological parameters between two body surfaces of an animal indicates that one body part might be practically implemented by another to measure the body temperature non-invasively.

Pearson correlation showed RT has a positive association with all IRT measures, including maximum and average values, where the highest association was observed between REM and RT (moderate correlation coefficients, r > 0.60). Previous studies demonstrated that eye temperature is a good indicator of animal health [14,44]. However, other studies revealed that the association of the mean IRT of eye and rectal temperature is low (correlation coefficients, r < 0.50) [42,45].

Although IRT changes frequently with environmental changes, as a non-invasive method, it is essential to develop a correlation with RT. Previous studies have applied a range of statistical approaches to develop rectal temperature assessment models in animals using their infrared temperature of different body surfaces, including linear regression, multiple linear regression, and stepwise linear regression [1,15,16,17,18]. The use of these techniques highlights ongoing efforts to identify the most reliable body regions and analytical strategies for accurately predicting rectal temperature from their infrared temperature. In this study, simple linear regression analysis was employed to develop a rectal temperature assessment model from the IRT of different body surfaces in goats for the tropical area where environmental temperature and relative humidity are comparatively high. We included data from the goats that did not show any signs of health deviations. Pearson correlation analysis reported that the highest correlation was between the REM and RT (r = 0.63), FHM and RT (r = 0.59), and REarM and RT (r = 0.57), consequently.

Simple linear regression analysis showed that all the assessment equations of REM, FHM, and REarM were significant (*p* < 0.05), which indicated that the goat surface temperatures measured by IRT could be used to assess rectal temperature. Here, we included only REM, FHM, and REarM because they exhibited the strongest association with RT, and they represent the rectal temperature most and least affected by environmental factors [1]. Thus, the correlation of this statistical model is relatively low as the value of R^2^ is less than 0.5, and REM and REarM showed moderate correlation with RT. Whenever Wiedemann et al. (2006) and Kunkle et al. (2004) reported a moderate correlation between IRT and RT in animals, it could be used as an alternative [46,47]. Therefore, as infrared thermography is a non-invasive technique to assess body temperature and can detect real-time changes in the animal body surfaces [48,49], it increases its acceptability in animals. In addition, we can use the maximum value of the IRT of the right eye and right ear to detect body temperature in goats, especially right eye temperature, as it exhibited the strongest association with rectal temperature, and apply the linear regression analysis model to measure rectal temperature.

## 5. Conclusions

The IRT of different body surfaces showed a positive association with RT. The highest association was observed between the maximum IRT of the right eye and rectal temperature. In addition, the IRT of the right eye and the left eye did not show any difference. Thus, the RT can be estimated accurately through the IRT of REM, and the IRT of REM could be a valid method to estimate the core temperature of goats under farm conditions remotely and noninvasively. However, further research is warranted to investigate the automatic record of eye IRT of goats in a commercial goat farm.

## Figures and Tables

**Table 1:** Descriptive statistics of rectal temperature (RT, °C) and infrared temperatures (IRT, °C) of a goat’s external body surfaces. Data evaluated from 84 goats.

Temperature of Body Parts	N	Mean	SD	Minimum	Maximum
Rectal Temperature	82	39.0	0.46	37.8	40.1
Right Eye Maximum IRT	84	40.6	0.90	38.7	42.8
Right Eye Average IRT	84	39.5	1.11	36.0	42.0
Left Eye Maximum IRT	84	40.7	0.83	38.3	42.7
Left Eye Average IRT	84	39.5	1.11	36.5	42.1
Forehead Maximum IRT	84	39.5	1.14	36.6	42.5
Forehead Average IRT	84	37.2	0.78	36.4	40.9
Right Coronary Band Maximum IRT	84	38.2	1.07	36.5	41.1
Right Coronary Band Average IRT	84	37.1	0.62	36.5	39.9
Left Coronary Band Maximum IRT	84	38.2	1.05	36.5	41.4
Left Coronary Band Average IRT	83	37.0	0.48	36.4	38.7
Right Ear Maximum IRT	84	39.5	1.16	37.2	42.2
Right Ear Average IRT	84	37.7	0.87	36.6	41.5
Left Ear Maximum IRT	84	39.7	1.09	37.4	42.1
Left Ear Average IRT	84	37.9	0.97	36.6	41.1
Right Shoulder Maximum IRT	84	38.8	1.22	37.2	42.2
Right Shoulder Average IRT	84	37.3	0.59	36.5	40.1
Left Shoulder Maximum IRT	84	38.9	1.16	37.1	41.8
Left Shoulder Average IRT	84	37.4	0.66	36.5	40.6
Mucous Membrane of Mouth Maximum IRT	84	41.1	0.89	38.9	42.9
Mucous Membrane of Mouth Average IRT	84	39.8	1.23	37.1	42.2

**Table 2:** Estimation of paired difference in the infrared temperature (IRT, °C) of right to left body surfaces of the same organ.

Infrared Temperatures (IRT)	Mean	Standard Error of Mean	*p*-Value
Right vs. Left Eye Maximum IRT	−0.08	0.06	0.160
Right vs. Left Eye Average IRT	0.03	0.09	0.780
Right vs. Left Coronary Band Maximum IRT	0.00	0.08	0.955
Right vs. Left Coronary Band Average IRT	−0.02	0.03	0.522
**Right vs. Left Ear Maximum IRT**	−0.19	0.07	0.011
Right vs. Left Ear Average IRT	0.03	0.09	0.780
Right vs. Left Shoulder Maximum IRT	−0.17	0.11	0.110
**Right vs. Left Shoulder Average IRT**	−0.13	0.03	0.000

Here, the bold text indicates the pair of body parts of goats with a significant difference.

**Table 3:** Pearson correlation between rectal temperature (RT) and maximum infrared temperature (IRT) of the goat’s different body surfaces. Correlations with coefficients > 0.75 were classified as high, those between 0.36 and 0.70 as moderate, and those <0.35 as low.

	RT	REM	LEM	FHM	RCBM	LCBM	REarM	LEarM	RSM	LSM
REM	0.63									
LEM	0.55	0.82								
FHM	0.59	0.63	0.63							
RCBM	0.43	0.56	0.46	0.46						
LCBM	0.56	0.60	0.58	0.51	0.73					
REarM	0.57	0.72	0.66	0.76	0.55	0.61				
LEarM	0.45	0.65	0.59	0.63	0.54	0.59	0.83			
RSM	0.43	0.60	0.58	0.62	0.51	0.57	0.69	0.65		
LSM	0.51	0.62	0.59	0.52	0.45	0.54	0.63	0.64	0.67	
MMM	0.54	0.65	0.60	0.56	0.53	0.55	0.71	0.66	0.55	0.61

Non-bold—*p* = 0.000. Where, RT = rectal temperature, REM = right eye maximum IRT, LEM = left eye maximum IRT, FHM = forehead maximum IRT, RCBM = right coronary band maximum IRT, LCBM = left coronary band maximum IRT, REarM = right ear maximum IRT, LEarM = left ear maximum IRT, RSM = right shoulder maximum IRT, LSM = left shoulder maximum IRT, and MMM = mucous membrane of mouth maximum IRT.

**Table 4:** Pearson correlation between rectal temperature (RT) and average infrared temperature (IRT) of the goat’s different body surfaces. Correlations with coefficients > 0.75 were classified as high, those between 0.36 and 0.70 as moderate, and those <0.35 as low.

	RT	REA	LEA	FHA	RCBA	LCBA	REarA	LEarA	RSA	LSA
REA	0.59									
LEA	0.55	0.71								
FHA	0.47	0.49	0.49							
RCBA	0.46	**0.35**	0.39	0.61						
LCBA	0.45	0.40	0.43	0.60	0.75					
REarA	0.48	0.55	0.49	0.51	0.35	0.44				
LEarA	0.54	0.53	0.53	0.56	0.52	0.57	0.62			
RSA	0.38	0.53	0.54	0.57	0.51	0.56	0.57	0.66		
LSA	0.38	0.49	0.52	0.56	0.44	0.52	0.58	0.70	0.74	
MMA	0.48	0.51	0.41	**0.33**	**0.35**	0.40	0.53	0.55	0.47	0.43

Non-bold—*p* = 0.000, Red—*p* = 0.001, and bold—*p* = 0.002. Where, RT = rectal temperature, REA = right eye average IRT, LEA = left eye average IRT, FHA = forehead average IRT, RCBA = right coronary band average IRT, LCBA = left coronary band average IRT, REarA = right ear average IRT, LEarA = left ear average IRT, RSA = right shoulder average IRT, LSA = left shoulder average IRT, and MMA = mucous membrane of mouth average IRT.

## Data Availability

The original contributions presented in this study are included in the article. Further inquiries can be directed to the corresponding authors.
